# Persistent *Borrelia burgdorferi* protein antigen in CD16 ^+^ monocytes is associated with monocyte-skewed immune dysregulation in post-treatment Lyme disease syndrome (PTLDS)

**DOI:** 10.64898/2026.09.24.26363601

**Published:** 2026-10-01

**Authors:** Bruce K. Patterson, Edgar B. Francisco, Nicolas Castaño, Kwat Medetgul-Ernar, Christopher Beaty, Kaeden Beaty, Patricia A. Felder, Ram Yogendra

## Abstract

Post-treatment Lyme disease syndrome (PTLDS) is characterized by persistent symptoms after antibiotic therapy for *Borrelia burgdorferi* infection. The mechanisms underlying these symptoms remain incompletely understood and have been attributed to ongoing low-level infection, autoimmunity, or immune dysregulation driven by residual bacterial antigens. We investigated whether *Borrelia burgdorferi* protein antigen persists in long-lived monocyte subsets in PTLDS patients and is associated with broader immune dysregulation. CD14 ^⁺^ CD16 ^⁺^ monocytes from 10 patients with PTLDS were analyzed by mass spectrometry; plasma from all 10 was profiled on a 14-plex cytokine panel against 50 healthy donors; PBMCs from 6 patients (3 antigen-positive and 3 antigen-negative) underwent RT-qPCR and bulk RNA-seq with immune-compartment profiling. FlaB and OspC were detected in CD14 ^⁺^ CD16 ^⁺^ monocytes months to years after treatment, whereas *Borrelia burgdorferi* transcript was not detected. Plasma IFN-γ exceeded the healthy donor median in all 10 participants. Antigen-positive samples showed an antigen-associated inflammatory transcriptional profile, including increased TNFSF14/LIGHT, GSDMD, GBP5, and KCNJ2 expression, together with a compartment-level pattern of monocyte-skewed myeloid reconfiguration, higher neutrophil-related features, and selective T-cell-state changes. These findings link residual *Borrelia burgdorferi* protein in CD14 ^⁺^ CD16 ^⁺^ monocytes to monocyte-skewed immune remodeling in PTLDS, paralleling observations in long COVID and supporting a model in which post-infectious syndromes are sustained by antigen-fueled immune remodeling rather than ongoing infection.

**Importance:**

Many people treated for Lyme disease go on to experience fatigue, pain, and difficulty thinking for months or years, and the reason is unknown. Persistent infection has been a leading explanation, yet longer courses of antibiotics do not help. We found proteins from *Borrelia burgdorferi* , the bacterium that causes Lyme disease, inside long-lived blood monocytes of patients with lasting symptoms, years after treatment and with no sign that the bacterium was still active. Patients carrying these proteins showed a distinct inflammatory immune profile. These findings point to leftover bacterial material, rather than ongoing infection, as a possible driver of persistent symptoms, echo observations in long COVID, and argue that treatments directed at the immune response deserve study.

## Full Text Availability

The license terms selected by the author(s) for this preprint version do not permit archiving in PMC. The full text is available from the preprint server.

